# Metabolic and immune profiles of 1-year and 2.5+ year-old white leghorn roosters following intramuscular lipopolysaccharide injection

**DOI:** 10.3389/fvets.2025.1547807

**Published:** 2025-02-19

**Authors:** Kayla M. Elmore, Elizabeth A. Bobeck

**Affiliations:** Department of Animal Science, Iowa State University, Ames, IA, United States

**Keywords:** C-reactive protein, immune cell profile, immunometabolic assay, lipopolysaccharide, poultry

## Abstract

Aerosolized endotoxins such as Lipopolysaccharide (LPS), found in livestock environments, induce an inflammatory mediator cascade. Poultry are commonly exposed to LPS over the growth cycle; however, little is known regarding the cumulative impact of intramuscular LPS injection and its effects on immune cellular metabolism, pathway preferences, and clearance response. Utilizing a LPS model in chickens can offer insight into host immune responses and provide a better understanding of immune tolerance to this endotoxin and major component of Gram-negative bacteria. Therefore, the study objectives were to compare metabolic phenotypes and immune profiles of isolated peripheral blood mononuclear cells (PBMC) from two ages of adult White Leghorn roosters before and post-LPS injection. A total of 20 adult White Leghorn roosters aged 1 yr. or 2.5+ yrs. were randomly assigned to sterile saline or 1 mg/kg body weight LPS (*Escherichia coli* O55:B5, LPS) injected intramuscularly across 4 sites in breast and thigh muscles. Body weight was recorded before injections at baseline and 24 h post-injection (hpi). Cloacal temperature and blood collections were performed at baseline, 6 hpi, and 24 hpi. PBMC were isolated for Agilent Seahorse XF metabolic analysis and multicolor flow cytometry. Plasma was collected for a C-reactive protein (CRP) enzyme-linked immunosorbent assay. Statistical analysis was performed using the MIXED procedure with fixed effects of age, injection status, and age X injection interaction followed by Tukey–Kramer adjustment (SAS 9.4), with significance denoted at *p* ≤ 0.05. Aged roosters were found to have fewer CD3^+^CD8α^+^ T cells at baseline compared to younger roosters (*p* < 0.05) while generally displaying delayed immunometabolic changes post-LPS injection compared to younger roosters. Young roosters administered LPS had significantly reduced CRP at 6 hpi compared to control, while aged roosters significantly increased CRP production by 24 hpi (*p* < 0.05). Both ages responded similarly to inhibitory assays, suggesting that the ability to respond was not different based on age. Overall, results suggest adult roosters may respond differently to LPS injection based on age and immune cell presence, likely due to accumulated exposure to LPS in poultry environments.

## 1 Introduction

Common immune system challenges that poultry experience include environmental exposure to *Salmonella* and *Escherichia coli*, which are Gram-negative bacteria with lipopolysaccharide (LPS) in their outer membranes. LPS initiates an inflammatory response by promoting macrophage and dendritic cell activity, which leads to cytokine release and immune cell activation (1). Immune cell activation relies on metabolic state switching, which is crucial for proper function (2). During activation, immune cells shift to aerobic glycolysis, known as the “Warburg effect” to meet energy demands, producing ATP faster than oxidative phosphorylation, though less efficiently (3, 4). This shift supports rapid energy production and enables immune activation, but also triggers physiological responses in poultry such as inflammation, fever, weight loss, reduced feed intake, and decreased egg production (5, 6).

Previous research has shown that the immune response to LPS varies based on factors such as age, sex, breed, prior exposure, and the method of LPS administration (7–10). Age plays a crucial role in immune response development. For example, naturally occurring antibody concentrations against bacterial infections, including LPS, have been shown to increase with age, as observed in 5- and 18-wk-old laying hens (11, 12). In addition to antibody production, age-related differences have also been observed in physiological responses to LPS. Using a 1 mg/kg intravenous LPS injection model in poultry, researchers observed that 3 and 5-wk-old broilers exhibited increased body temperatures that persisted past 15 h post-injection (7, 13), with 3-wk-old broilers showing a higher maximum body temperature compared to 5-wk-old broilers (13). Genetic factors have also been shown to influence physiological outcomes. Using a 2 mg/kg intramuscular LPS injection model, LPS-induced weight loss and genotype-specific temperature profiles within the first 24 h post-LPS injection 12-wk-old cockerels and 18-wk-old pullets (14, 15). Additionally, repeated LPS exposure was shown to modulate immune tolerance, where a second 1 mg/kg intravenous LPS dose after two or seven days post-injection reduced fever responses and altered interleukin-6 (IL-6) concentrations in 3 and 5-wk-old Ross broilers (13). At a cellular level, metabolic adaptation in response to LPS provides further insight into immune response. Previous *in vitro* studies have demonstrated that LPS challenge can enhance energy metabolism through glycolysis, suggesting a shift to a more metabolically active state to support immune function (1, 16, 17). However, despite these findings, there is still limited research on how cellular metabolism changes under stress conditions, such as LPS injection, in avian models. Understanding these metabolic shifts is key to determining how immune cells allocate energy during an immune challenge and whether these energetic demands vary with age.

One way to address this gap is through real-time metabolic and flow cytometric analyses of chicken peripheral blood mononuclear cells (PBMC). These assays would reveal the immune cell populations before and after LPS challenge, provide insight into the metabolic demands of these cells and how their energetic requirements evolve during the immune response and LPS clearance. Another strategy is to use systemic stress biomarker assays to confirm LPS-induced inflammation and assess the immune response potential. LPS binding to myeloid differentiation factor 2 and Toll-like receptor 4 heterodimers on immune cells induces the production and release of various pro-inflammatory cytokines, including interleukin-1, IL-6, and interferon-gamma (18–20). This cytokine release drives hepatocytes to produce and secrete C-reactive proteins (CRP) into the bloodstream, facilitating phagocytosis in response to infection and inflammation (21–23). Therefore, the current study measured circulating CRP levels using enzyme-linked immunosorbent assays (ELISA) to assess the inflammatory response stimulated by intramuscular LPS injections.

Integrating these three analyses, immune cell profiling, immunometabolic shifts, and systemic inflammation confirmation, provides a comprehensive approach to further understanding the immune response to an LPS challenge in poultry. The present study aimed to investigate metabolic phenotypes and cell profiles of PBMC of two age groups (~1 yr. and ~ 2.5 yrs. of age) of adult White Leghorn roosters before and after intramuscular LPS injection with *Escherichia coli*. These birds had been raised in the same facility and conditions for the duration of their life, and therefore represented a cohort for study with the differing factor of age. Additionally, plasma CRP concentrations, a key biomarker for systemic stress and inflammation, were evaluated following the LPS challenge to further assess the immune response and its variation with age.

## 2 Materials and methods

### 2.1 Animals, housing, and treatments

All procedures were approved by the Iowa State University Institutional Animal Care and Use Committee #22–113. Twenty total White Leghorn roosters were enrolled from an existing flock maintained at Iowa State University’s Robert T. Hamilton Poultry Teaching & Research Facility (Ames, IA). All enrolled birds were adults at the time of the experiment; however, will be separated into two cohorts: ~1 yr. (~55 wk., young) and ~ 2.5+ yrs. (~133 wk., aged), for ease of discussion. Only roosters were included in this study due to the unavailability of an age-matched cohort of adult females. All birds were raised and housed under the same conditions to ensure that age was the primary difference factor. Roosters were housed in individual hanging cages with *ad libitum* access to feed and nipple waterers. Twelve young roosters and 8 aged roosters were randomly assigned to one of two injection treatments, resulting in a 2 × 2 factorial of age (young or aged) by injection (CON or LPS). Each bird received an intramuscular injection of either 1 mg/kg body weight dosage of lipopolysaccharide (LPS) derived from *Escherichia coli* O55:B5 (Sigma-Aldrich, MO, USA) or an equivalent amount of 0.9% sterile saline as a control (CON). Intramuscular injection was chosen as the method of administration as birds were exposed to LPS while avoiding direct intravenous administration and preventing aerosolization associated with intranasal delivery, which could have impacted other research birds housed in the same facility. Each treatment dose was evenly divided across four sites, with ¼ of the injection administered to both the right and left sides of the breast and thigh muscles. Individual body weight and cloacal temperature data were collected to monitor physiological responses to injection. Body weight was measured immediately before injection (baseline) and 24 h post-injection (hpi), while cloacal temperature was recorded at all three timepoints (baseline, 6 hpi, and 24 hpi).

### 2.2 Peripheral blood mononuclear cell isolation

At all timepoints, approximately 750 μL-1 mL of blood was collected from the brachial vein of all roosters using heparinized syringes and collection tubes. PBMC were isolated using a high specific density gradient and processed following similar published methods (24–26). To limit granulocyte contamination, 3 mL of Histopaque 1077 was carefully layered onto 3 mL of Histopaque 1119 (Sigma Aldrich, MO, USA). Whole blood was diluted 1:1 with sterile, filtered phosphate-buffered saline (PBS, pH = 7.4), carefully layered onto Histopaque 1077, and centrifugation (650 x g for 35 min with minimal acceleration and brakes). Plasma and PBMC were collected from the Histopaque 1077 interface. Plasma was frozen at-80°C for CRP ELISA. Each PBMC sample was washed in PBS twice and resuspended in 1 mL of Seahorse assay media (pH 7.4, 37°C; Agilent, CA, USA). Each PBMC sample was enumerated using a hemocytometer after dilution to the same concentration with sterile, filtered PBS and trypan blue for viability assessment and enumerated by hemocytometer for use within the Seahorse XFe96 Analyzer (Agilent, CA, USA). Following use in Seahorse XF metabolic assays, 500 μL of a cryoprotective solution containing 85% chicken serum (Thermo Fisher Scientific, MA, USA) and 15% dimethyl sulfoxide (DMSO; Sigma-Aldrich, MO, USA) was added to each PBMC. All samples were frozen at-80°C prior to flow cytometric analysis in order to run all samples at the same time.

### 2.3 Flow cytometric analysis

PBMC from all three timepoints were thawed and washed in RPMI-1640 medium (Cytiva-Hyclone, Logan, UT) and resuspended in 500 μL sterile PBS (Corning, Corning, NY). Each PBMC sample was enumerated by hemocytometer and trypan blue staining before being aliquoted evenly across 6 polystyrene tubes (Corning, Corning, NY). The panel used in this study consisted of innate immune cell and lymphocyte markers: anti-chicken monocyte/macrophage PE (clone KUL01; mouse IgG1κ), anti-chicken cluster of differentiation (CD3) Pacific Blue™ (clone CT-3; mouse IgG1κ), anti-chicken CD1.1 FITC (clone CB3; mouse IgG1κ), anti-chicken CD4 PE/Cy7 (clone CT-4; mouse IgG1κ), anti-chicken CD8α Alexa Fluor® 700 (clone CT-8; mouse IgG1κ; Southern Biotech, Birmingham, AL). To address nonspecific binding, fluorescence-minus-one controls (FMO) and corresponding isotype controls were included in the staining procedure. The first tube of each sample contained a single mixture of all extracellular markers. Using the FMO method, subsequent tubes contained a similar mixture with one marker substituted by its corresponding isotype control. Isotype controls were diluted 1:10 in PBS for all staining. For each primary stain mixture, 0.4 μL antibody and 0.2 μL diluted isotype were added to 50 μL PBS for each sample. 50 μL of each primary stain mix was added to corresponding aliquoted sample tubes. All tubes were incubated on ice in the dark at 4°C for 30 min. Following staining, cells were washed, resuspended in 250 μL PBS, and analyzed on a BD FACSCanto™ cytometer (BD Biosciences, San Jose, CA). FlowJo version 10.5.0 software was used to identify individual cell populations (BD Biosciences, San Jose, CA). Singlet-live cell populations were used to identify monocyte/macrophage^+^ and CD3^+^ cells, while CD3^+^ populations were further gated to identify CD1.1^+^, CD4^+^, and CD8α^+^ cells.

### 2.4 Seahorse XF metabolic assays

Following PBMC isolation, fresh PBMC were plated into 96 well Seahorse XF cell culture microplates (Agilent, CA, USA) in quadruplicate and at a density of 200,000 cells/well for use in the Real-Time ATP Rate Assay and Glycolytic Rate Assay kits within the Seahorse XFe96 Analyzer (Agilent, CA, USA). Agilent User Guides were followed to perform all metabolic assays. Briefly, the Real-Time ATP Rate Assay evaluates total adenosine triphosphate (ATP) production alongside specific contributions from glycolysis and mitochondrial oxidative phosphorylation. This assay uses injections of metabolic inhibitors, namely oligomycin and a 1:1 mixture of rotenone and antimycin A (Rot/AA). Oligomycin inhibits ATP synthase in the mitochondrial electron transport chain (ETC), while Rot/AA targets complexes 1 (NADH oxidoreductase) and 3 (cytochrome bc1) of the ETC. The assay then quantifies glycolytic ATP (glycoATP), derived from the glycolytic pathway converting glucose to lactate, and mitochondrial ATP (mitoATP) from oxidative phosphorylation, with total ATP being the sum of glycoATP and mitoATP rates. In contrast, the Glycolytic Rate Assay measures real-time glycolytic rates, including compensatory and residual glycolysis, particularly after mitochondrial suppression by sequential injections of Rot/AA and 2-deoxy-D-glucose (2-DG), a glucose hexokinase inhibitor. Following baseline measurements (basal proton efflux rate and basal glycolysis), Rot/AA is injected, leading to mitochondrial respiration inhibition and forced glycolytic switch to meet energic demands. This allows for the estimation of compensatory glycolysis. Then, glycolysis is inhibited through the competitive binding of glucose hexokinase from 2-DG, which stops glycolytic acidification and allows for the inclusion of other sources of extracellular acidification, not attributed to glycolysis or the TCA cycle in the calculation of post-2-DG acidification. Wave software (version 2.6.1) monitors shifts in media pH, oxygen consumption, and hydrogen ion production throughout both assays.

### 2.5 C-reactive protein assay

Frozen 1:1 plasma to PBS samples were thawed and diluted 16,000-fold, a previously determined optimal C- reactive protein (CRP) concentration. All samples and standards were plated in duplicate at 100 μL/well on pre-coated plates, and the remaining protocols outlined by the Chicken CRP ELISA kit were followed (MyBioSource, San Diego, CA). O.D. absorbance was read at 450 nm using a microplate reader (Agilent, Santa Clara, CA).

### 2.6 Statistical analysis

All data were analyzed using the following model:


$$Y_{ijk}=\mu +A_{i}+I_{j}+{\left(A\times I\right)}_{ij}+\varepsilon^{ijk}$$

where y^ijk^ is the observed effect at each timepoint, 𝜇 is the overall mean value, A_i_ is the age main effect at the 𝑖^𝑡ℎ^ level (𝑖 = 2; Young or Aged), I_j_ is the injection main effect at the 𝑗^𝑡ℎ^ level (𝑗= 2, CON or LPS), (A x I)_ij_ is the interaction of age and injection, and ϵ_ijk_ is the random error term for each measurement. The UNIVARIATE procedure was used to identify outliers in each data set. No more than two outliers exceeding 2.5 standard deviations from the treatment mean were removed from the Seahorse metabolic data sets for the young rooster cohort at baseline, while all other data sets contained no outliers. Outlier removal was performed on individual measures, which included multiple repeated measures at the same timepoint, while all other data sets had no outliers. All data were analyzed using a mixed linear model with Tukey–Kramer adjustment to account for multiple comparisons (PROC MIXED, SAS 9.4, Cary, NC). The individual bird was the experimental unit. The random effect of the tube was used for flow cytometric data, and the random effect of rooster was used for all other data. Fixed effects of age, injection status, and the age X injection interaction were analyzed for all measures at each timepoint (baseline, 6 hpi, and 24 hpi). Least square means (LSMeans) and standard error (SEM) were reported, with significance observed at Tukey–Kramer adjusted *p* ≤ 0.05. The effect of time was initially included in the model, but due to resulting complex interaction effects, only data within timepoint is reported.

## 3 Results

### 3.1 Physiological outcomes

Before LPS injection, young (~1 yr) roosters weighed 0.53 kg more than aged (+2.5 yrs) roosters (*p* < 0.0001, Table 1). Aged roosters had a lower average baseline cloacal temperature by 0.55°C compared to young roosters (*p* = 0.01). At 6 hpi, aged roosters inoculated with LPS had significantly lower cloacal temperatures by 0.92–1.22°C compared to all other roosters (*p* = 0.01). No changes in temperature or starting BW were found at 24 hpi.

**Table 1:** Body weight and temperature by rooster age ± 1 mg/kg intramuscular LPS injection at all timepoints.

Measure	Young		Aged		Adj. *p*-value		
	CON	LPS	CON	LPS	Age	Trt^1^	Age x Trt^1^
BW (kg)							
Baseline	2.38 ± 0.05^a^	-	1.85 ± 0.06^b^	-	<0.0001	-	-
24 hpi	2.42 ± 0.07	2.26 ± 0.07	1.84 ± 0.08	1.78 ± 0.08	<0.0001	0.16	0.47
ΔBW for 24 hpi	−0.04 ± 0.01	−0.04 ± 0.01	−0.02 ± 0.02	−0.04 ± 0.02	-	-	-
Temperature (°C)							
Baseline	41.33 ± 0.12^a^	-	40.78 ± 0.13^b^	-	0.01	-	-
6 hpi	41.48 ± 0.18^a^	41.62 ± 0.18^a^	41.32 ± 0.21^a^	40.40 ± 0.21^b^	0.002	0.06	0.01
24 hpi	41.06 ± 0.16	41.32 ± 0.16	40.93 ± 0.19	40.91 ± 0.19	0.14	0.51	0.45
ΔTemperature for 6 hpi	0.21 ± 0.09	0.23 ± 0.17	0.49 ± 0.16	−0.33 ± 0.16	-	-	-
ΔTemperature for 24 hpi	−0.20 ± 0.09	−0.06 ± 0.06	0.10 ± 0.16	0.18 ± 0.16	-	-	-

Data represents the mean ± SEM (*n* = 12 young and 8 aged roosters at baseline, *n* = 6 young and 4 aged roosters/treatment at 6 hpi, and *n* = 6 young and 4 aged roosters/treatment at 24 hpi). Different letter superscripts within a timepoint are significantly different (Adjusted *p* ≤ 0.05). ^1^Trt, Injection main effect.

### 3.2 PBMC profile

**Figure 1:**
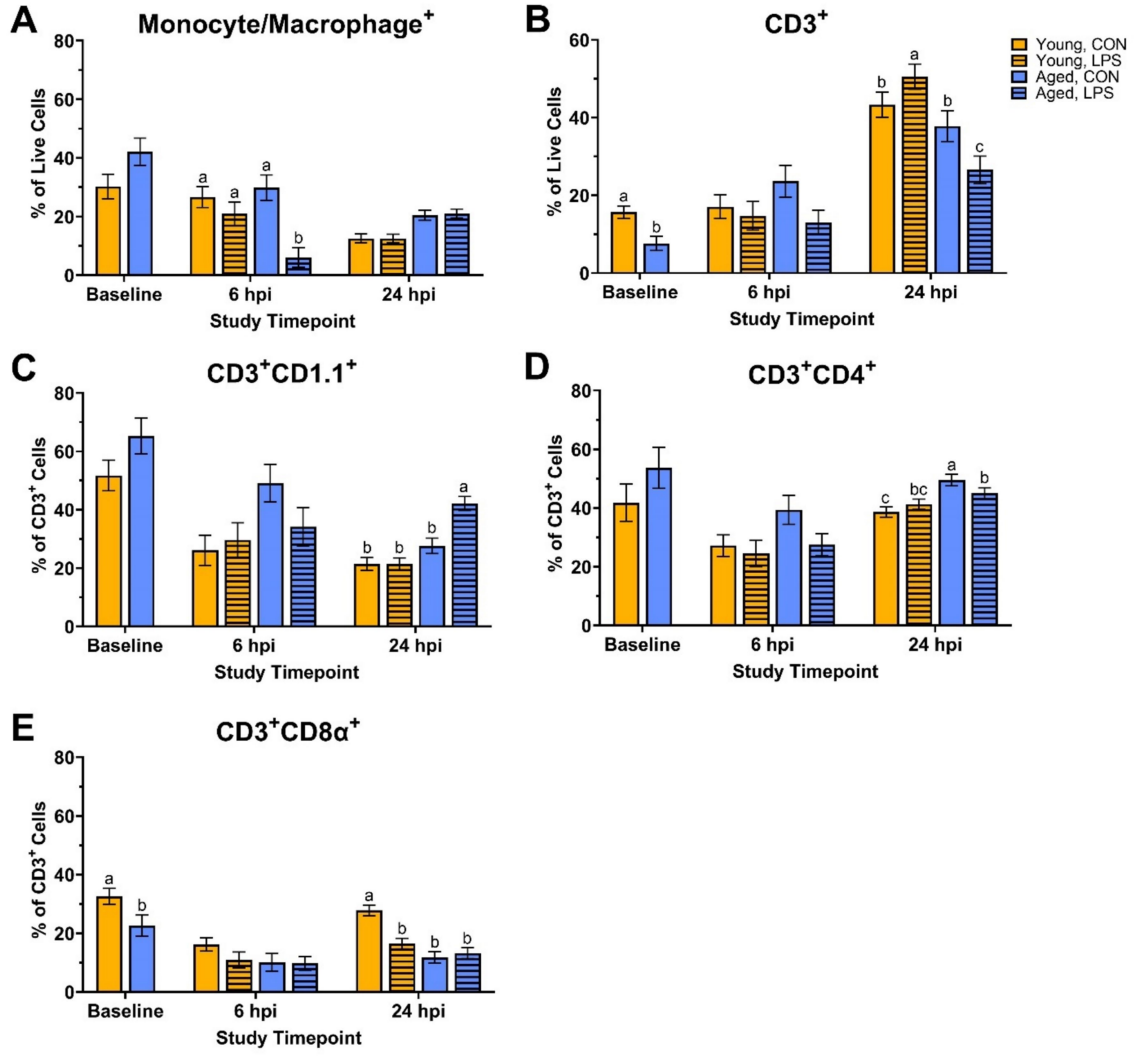
Percentages of **(A)** Monocyte/Macrophage^+^, **(B)** CD3^+^, **(C)** CD3^+^ CD1.1^+^, **(D)** CD3^+^ CD4^+^, and **(E)** CD3^+^ CD8α*^+^* cells isolated from peripheral blood mononuclear cells of White Leghorn roosters (young and aged) ± 1 mg/kg intramuscular LPS injection at baseline, 6 hpi, and 24 hpi. Data represents the mean and SEM (*n* = 12 young and 8 aged roosters at baseline, *n* = 6 young and 4 aged roosters/treatment at 6 hpi, and *n* = 6 young and 4 aged roosters/treatment at 24 hpi). Different letter superscripts within a timepoint are significantly different (Adjusted *p* ≤ 0.05).

At baseline, young roosters had 51.2% more CD3^+^ cells, and specifically, 30.5% more CD3^+^CD8α^+^ cells compared to aged roosters (*p* = 0.01 and *p* = 0.04, respectively; Figures 1B,E). No other differences in monocyte/macrophage^+^, CD3^+^CD1.1^+^, and CD3^+^CD4^+^ cells were found at baseline (*p* > 0.05; Figures 1A,C,D). At 6 hpi, aged LPS-administered roosters had significantly fewer monocyte/macrophage^+^ cells than all other groups, and this population recovered by 24 hpi (71.0–79.7%; *p* = 0.02, Figure 1A). No other queried populations were different at 6 hpi.

At 24 hpi, aged LPS-administered roosters had 29.8% fewer CD3^+^ cells than their counterpart control and 47.5% fewer than young LPS-administered roosters (*p* = 0.01, Figure 1B). A similar trend was observed in CD3^+^CD4^+^ cells, where aged LPS-administered roosters had 9.0% fewer CD3^+^CD4^+^ cells than aged control roosters (*p* = 0.02, Figure 1D), while young roosters remained unchanged. Aged LPS-administered roosters had 34.4% more CD3^+^CD1.1^+^ cells than their control counterparts, with young roosters showing no significant changes at 24 hpi (*p* = 0.001, Figure 1C). At 24 hpi, LPS reduced CD3^+^CD8α^+^ cells by 40.8% compared to young control roosters, with no differences observed in aged roosters due to LPS (*p* < 0.0001, Figure 1E).

### 3.3 Seahorse immunometabolic phenotype

**Figure 2:**
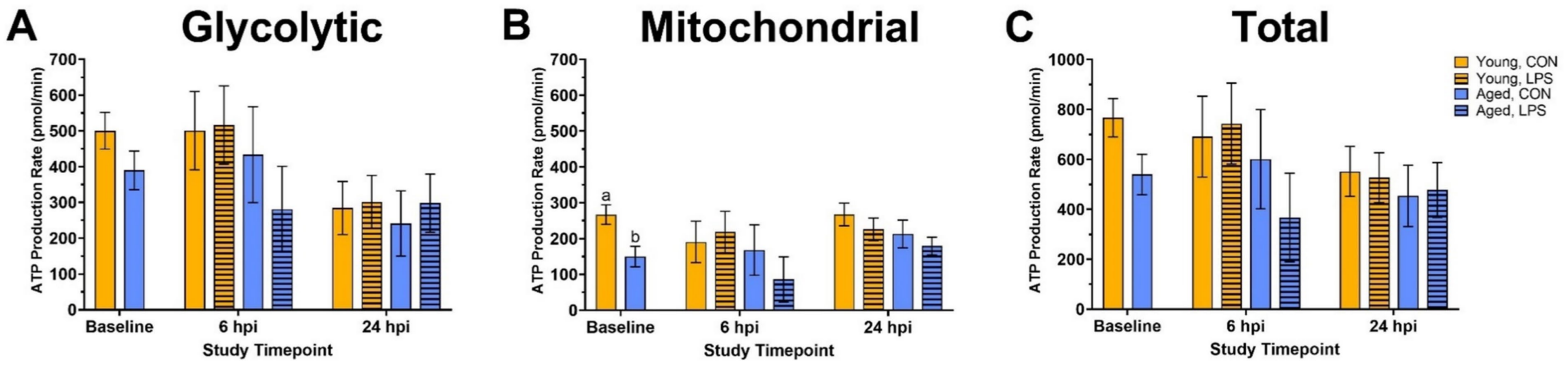
**(A)** Glycolytic, **(B)** mitochondrial, and **(C)** total ATP production of peripheral blood mononuclear cells isolated from 2 ages of White Leghorn roosters ±1 mg/kg intramuscular LPS injection at baseline, 6 hpi, and 24 hpi. Note that the y-axis is on a larger scale in panel **(C)**. Data represent the mean and SEM (*n* = 10 young and 8 aged roosters at baseline, *n* = 6 young and 4 aged roosters/treatment at 6 hpi, and *n* = 6 young and 4 aged roosters/treatment at 24 hpi). Different letter superscripts within a timepoint are significantly different (Adjusted *p* ≤ 0.05).

**Figure 3:**
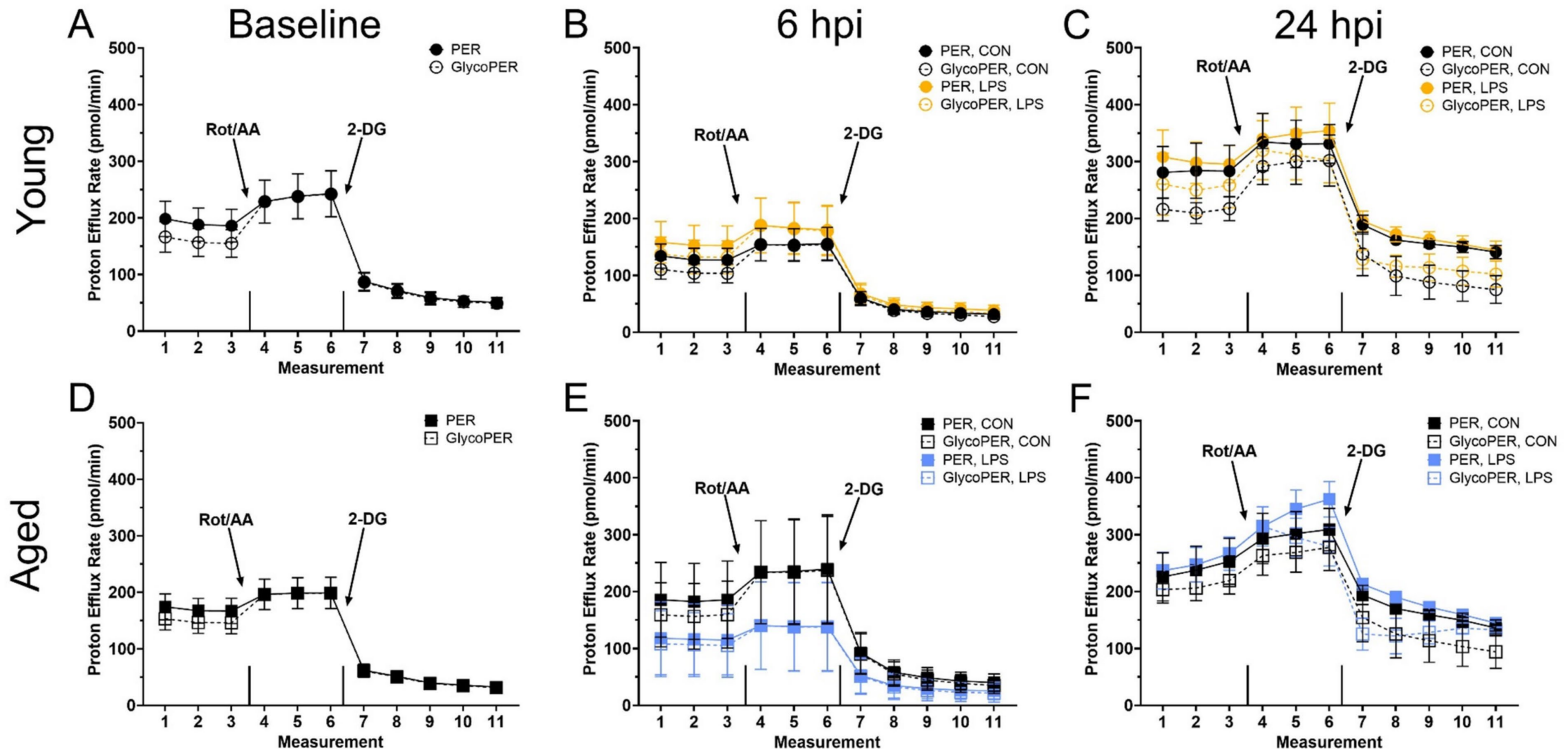
The basal proton efflux rate (PER) and glycolytic proton efflux rate (glycoPER) potential of peripheral blood mononuclear cells from **(A–C)** Young and **(D–F)** Aged roosters ±1 mg/kg intramuscular LPS inoculation at baseline, 6 hpi, and 24 hpi. Data represent the mean ± SEM (*n* = 12 young and 8 aged roosters at baseline, and n = 6 young and 4 aged roosters at 6 hpi and 24 hpi); no significant differences were found within any timepoint. This assay measures real-time glycolysis, including compensatory and residual glycolysis, by tracking extracellular acidification. After basal PER and glycolysis are measured, rotenone/antimycin A (Rot/AA) inhibits mitochondrial respiration, forcing a glycolytic shift to estimate compensatory glycolysis. This is followed by 2-deoxy-D-glucose (2-DG), which inhibits glycolysis, allowing differentiation of acidification sources beyond glycolysis and the TCA cycle.

Before LPS injection, mitochondrial ATP production increased in young roosters by 43.9% at baseline compared to aged (*p* = 0.01, Figure 2B), while glycolytic and total ATP production remained similar across ages (*p* > 0.05; Figures 2A,C). In addition, no differences in basal glycolysis, basal PER, compensatory glycolysis, or post-DG acidifications due to age were observed at baseline (*p* > 0.05, Figures 3A,D). At both 6 and 24 hpi, age, injection, and their interaction did not significantly affect ATP production rates nor glycolytic rate (*p* > 0.05; Figures 2A–C, 3A–C).

### 3.4 Plasma C-reactive protein

**Figure 4:**
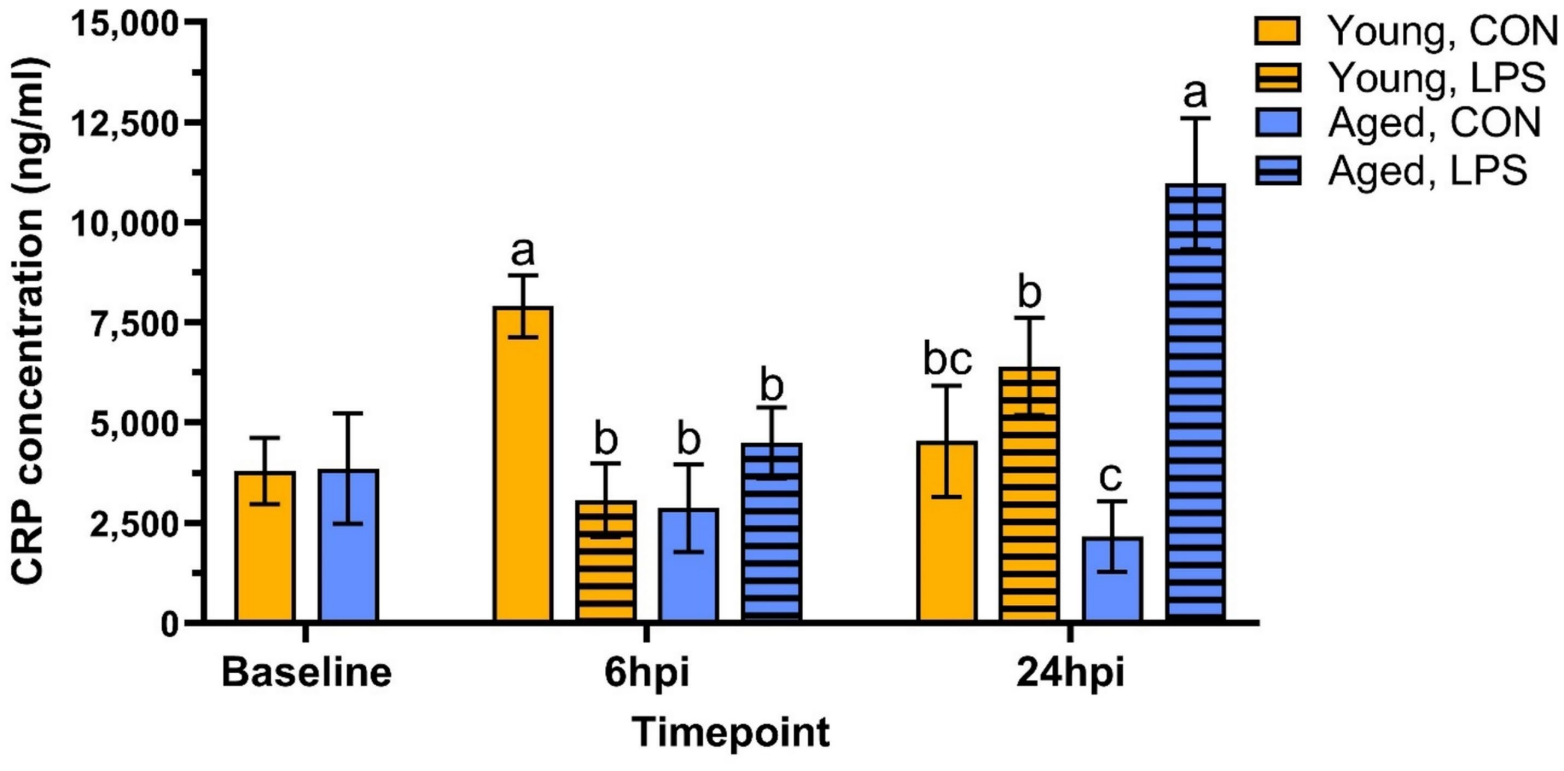
Plasma C-reactive protein concentration from genetic line roosters ±1 mg/kg intramuscular LPS injection at baseline, 6 hpi, and 24 hpi. Data represent the mean and SEM (*n* = 12 young and 8 aged roosters at baseline, n = 6 young and 4 aged roosters/treatment at 6 hpi, and *n* = 6 young and 4 aged roosters/treatment at 24 hpi). Different letter superscripts within a timepoint are significantly different (Adjusted *p* ≤ 0.05).

At baseline, CRP concentrations were not different due to age (*p* = 0.97, Figure 4). At 6 hpi, LPS significantly reduced CRP in young roosters by 61.3% compared to young control, while aged roosters remained similar (*p* = 0.01). A delayed effect was observed in old roosters, where LPS induced 80.3% more CRP production in aged roosters than their control counterpart (*p* = 0.02). In contrast, young roosters administered with LPS showed CRP levels that had returned to values similar to control.

## 4 Discussion

In the present study, the effects of intramuscular LPS injection on physiological and metabolic phenotypes were investigated in PBMC isolated from two ages of adult White Leghorn roosters. CRP concentration, a biomarker for systemic inflammation and stress, was also measured to understand further the LPS model used in this study.

Age-related differences in baseline physiological measures were expected. Body weight tends to decline with age due to the loss of lean body mass and bone density (27). In line with this, aged roosters in this study weighed less than their younger counterparts. Additionally, baseline cloacal temperatures were lower in aged roosters. Reduced metabolic rate, altered thermoregulation efficiency, and decreased cardiovascular function may contribute to lower body temperatures and are often associated with aging in mammals (28–31), while these observations are more variable in avian species. However, both age groups fell within the normal body temperature range for poultry (32). In addition, young and aged roosters showed distinct immune cell profiles and metabolic activity at baseline. Young roosters had significantly higher percentages of CD3^+^ and CD3^+^CD8α^+^ cells than aged roosters, indicating a more circulating T-cell presence in the younger birds. Similarly, previous research involving H&N and Dekalb Delta White Leghorn chickens showed the portion of peripheral blood CD3^+^ and CD8^+^ lymphocytes significantly decreased from 9-wk-old juvenile birds to 79- or 80-wk-old adult birds (33). This may suggest an age-related decline in immune function. Metabolically, young roosters had a greater mitochondrial ATP production at baseline than older birds, suggesting more efficient energy production via oxidative phosphorylation in younger birds. However, glycolytic ATP production and total ATP output remained comparable between the two age groups, suggesting that glycolysis may be less affected by age. Despite age-related differences observed in ATP production, young and aged birds responded similarly to the Rot/AA and 2-DG inhibitors within the Glycolytic Rate Assay, showing an equal capability to switch between fuel sources. We have seen this previously in a laying hen injection model (25).

Regarding systemic inflammation, plasma CRP concentrations did not differ between young and aged roosters before injection, indicating that baseline inflammation levels measured by CRP were similar across ages. This indicates systemic inflammatory states were stable despite age-related differences in immune cell profiles and metabolism. Additionally, as both groups of roosters were raised and housed under identical conditions at the same poultry farm, external factors such as diet, housing, and general management were controlled. Hens were not included because there was not two ages of hens nor an age-matched cohort in the research flock. Therefore, the main difference between the two age groups was their accumulated exposure to environmental LPS over time in the same facility. Together, these findings suggest that younger roosters exhibit a stronger T-cell profile and greater mitochondrial ATP production, while other immune and metabolic parameters remain consistent across age groups prior to LPS challenge.

At 6 hpi, aged LPS-administered roosters showed significantly lower cloacal temperatures, suggesting a blunted fever response that may be attributed to age. A similar hypothermic response has also been observed in 3- and 5-wk-old Ross broilers following intravenous LPS injection of 1 mg/kg bw (*Escherichia coli* O127: B8) but had transitioned into a fever response by 5 hpi (13). In contrast, body temperature increased from 2 hpi through 16 hpi following intravenous LPS injection (*Escherichia coli* O127:B8) at the same dosage in 5-week-old broilers (7). Using an alternative LPS administration route, a fever response was observed as early as 2 h post-intraperitoneal injection of 1 and 2.5 mg/kg bw LPS (*S. typhimurium*) doses in 34-d-old Brown Nick layers (34). In the same study, a fever response occurred between 4–12 h post-LPS injection at doses of 0.1–5 mg/kg bw in layers, with peak fever observed at 4 hpi (34). Therefore, given the discrepancy between these findings, the lack of a fever response in the current study was unexpected but not entirely surprising, as physiological responses to LPS vary greatly across studies. Further, the current study may have been due to several experimental factors, including the timing of temperature monitoring, LPS administration route, LPS isolate strain, and differences in bird age or genetic line. Additionally, the potential of chronic exposure to low levels of environmental LPS in these roosters may have induced immune tolerance and be another plausible explanation for the lack of a significant fever response in a single LPS challenge.

In comparing immune cell profiles, aged LPS-administered roosters had significantly fewer monocyte/macrophage^+^ cells than all other groups at 6 hpi, suggesting cell migration from circulation to affected tissues due to LPS (35). However, no significant differences were observed in the other immune cell populations across age or treatment groups at this time point. Metabolically, ATP production rates and glycolytic activity were not significantly impacted by age, injection, or their interaction at 6 hpi. This suggests that PBMC cellular energy output from mitochondrial and glycolytic pathways remained stable regardless of LPS challenge or age of the roosters. Interestingly, both PER and glycoPER also remained consistent from baseline to 6 hpi in both age groups, suggesting LPS injection did not hinder PBMC ability to successfully switch from mitochondrial respiration to glycolysis to meet energy demands and maintenance requirements during this period. The steady metabolic activity and lack of change following Rot/AA and 2-DG injections further support this finding. These results were unexpected, given LPS is well known for activating innate immune responses and driving metabolic reprogramming, where immune cells shift from oxidative phosphorylation to glycolysis to meet increased energetic demands following activation (1, 3, 4, 36). Therefore, we anticipated a significant increase in glycolytic ATP production, PER, and glycoPER in LPS-administered birds, regardless of age.

Birds already rely heavily on glucose as an energy source, as their typical resting homeostatic glucose is above 200 mg/dL, and they are glucagon, not insulin-responsive (37). Metabolic shifts may not be as drastic upon immune activation due to prior experience with LPS, which is highly likely in adult chickens as LPS is commonplace in poultry environments. Poultry also already have a glucose-rich environment perhaps quickly accessible for peripheral immune cell activation. Therefore, 6 hpi may not be an optimal timepoint to capture the expected PBMC immune cell profile and metabolic shifts post-LPS injection. It is possible that metabolic changes occurred at earlier or later time points, emphasizing the need for future studies with expanded sampling windows to better characterize immune cell metabolism following LPS challenge. Previously, we have compared PBMC metabolism within 22- and 96-wk-old commercial White Leghorn laying hens during a *Staphylococcus aureus* challenge model (25). The results align with the current rooster study, as no significant age-related differences in baseline metabolic potential were observed in adult hens. However, the age differences are not directly aligned with those in the current work. Further, the previous study also suggested hens may have remained metabolically stressed from transport and re-housing past a 72-h acclimation period through 3 d post-*Staphylococcus aureus* inoculation, where the PBMC did not alter glycolytic capacity in response to inhibitor drugs (25). In contrast, roosters in the present study were already housed long-term on-site, eliminating transport stress as a confounding factor. However, the lack of metabolic response to Rot/AA seen in the baseline through 24 hpi profiles may have several explanations. Firstly, it could indicate acute handling stress, although the birds were handled regularly for semen collection. Alternatively, PBMC may have already been operating at preferred or maximal capacity for anaerobic glucose production, as the general profiles in Figure 3 shows a typical reduction in baseline PER response to 2-DG, which was reduced by LPS at 6 hpi (as a trend) and recovered by 24 hpi.

Previous work comparing different poultry lines and ages further highlights the metabolic variability at baseline and during immune challenge (25, 38, 39). A direct comparison of 6-wk-old Ross 308 broiler chicks to >56-wk-old broiler breeders revealed that older birds relied more on oxidative metabolism (40). Similarly, the aged layers over 100 wk. had increased metabolic capacity as well as comparatively increased reliance on oxidative metabolism compared to the young layer chicks. However, the older laying hens over 100 wk. preferred glycolysis, which is consistent with the current findings. Both young and aged hens retained the capacity to respond to metabolic inhibitors, with aged birds showing a heightened capacity (40).

Serum CRP assays were utilized in the current study to further assess immune activation and systemic inflammation at baseline and post-LPS challenge. CRP plays a key role in innate immune response, as it repairs damaged tissue, recognizes pathogens, and facilitates their removal through the complement system and phagocytes (41, 42). Increasing levels of circulating CRP have been detected in broilers following bacterial infections [*Salmonella typhimurium* lipopolysaccharide (43)]. In the current study, plasma CRP levels varied between groups at 6 hpi. Interestingly, young control roosters had higher CRP concentrations than their LPS-administered counterparts, suggesting a more pronounced inflammatory response in unstimulated birds at this timepoint. In contrast, aged roosters showed no significant difference in CRP levels until 24 h, indicating that a faster systemic inflammatory response was more pronounced in the younger controls compared to LPS-administered birds. These results strengthen the need for future studies with extended sampling timepoints to understand systemic inflammation in adult birds following intramuscular LPS challenge. Additionally, measuring pro-inflammatory cytokine expression, such as interleukin-1 (IL-1), interleukin-6 (IL-6), and tumor necrosis factor-*α* (TNF-α), would be an alternative assay to investigate LPS-induced inflammation further. Using a similar LPS strain as the present study (*Escherichia coli* 055: B5), an intraperitoneally LPS dose of 1 mg/kg significantly increased IL-1, IL-6, and TNF-α in 20 and 27-d-old broilers (44).

By 24 hpi, immune responses of the roosters continued to differ based on age and LPS interaction. Initially observed differences in monocyte/macrophage cell populations at 6 hpi were resolved by 24 hpi, suggesting stabilization of an early innate immune response. However, LPS and age-related differences appeared within T-cell populations at 24 hpi. Young LPS-administered birds had more total CD3^+^ cells than their control counterparts, while aged LPS-administered roosters had fewer CD3^+^ and CD3^+^CD4^+^ cells than their control counterparts and young roosters. Interestingly, the inverse was observed in lipid antigen-presenting CD1.1^+^ cells, as aged LPS-administered roosters had more CD1.1^+^ cells than their control counterparts, while young roosters remained similar at 24 hpi. Meanwhile, young roosters had greater percentages of CD8α^+^ cytotoxic T-cells than their LPS-administered counterparts, which was not observed in aged roosters. These findings highlight age-related differences in immune activation, with young roosters having a stronger T-cell response, while aged roosters may have experienced impaired T-cell proliferation, delayed T-cell activation, and compensatory immune responses due to age-related immune decline and possible immunosenescence (45). However, further investigation is necessary to confirm peak shifts in cell populations and LPS clearance. For example, the increase in total CD3^+^ cells at 24 hpi may be the result of lymphatic migration, indicating 24 hpi was an appropriate timepoint to observe changes in CD3^+^ cell populations following intramuscular LPS injection, while alternative sampling timepoints may be more appropriate for remaining cell populations. Further, LPS-induced responses have been observed as early as 2 hpi, with peak responses recorded at 4 hpi in layers following intraperitoneally injection (34). Given intravenous and intraperitoneal LPS injections are known eliciting a rapid systemic immune response, selecting a timepoint after 6 hpi, but before 24 hpi, may be optimal to capture systemic responses that are delayed by the initial recruitment and activation of immune cells within the muscle tissue following intramuscular LPS injection. Regarding systemic inflammation at 24 hpi, plasma CRP concentrations were significantly elevated in aged LPS-administered roosters, while differences observed in young roosters at 6 hpi were resolved by 24 hpi. This may indicate a heightened inflammatory response in older birds at 24 hpi, whereas, in younger birds, CRP response to intramuscular LPS injection was not fully captured at the selected timepoints.

While physiological fever changes due to LPS injection were not observed within age groups in the current study, a notable change was observed in PBMC response post-LPS injection within the metabolic assays and immune profiles. Thus, LPS injection was adequate from a molecular standpoint as PMBC responded to metabolic challenges within the assays. This study provides insight into how metabolic activity, immune cell populations and CRP concentrations shift in PBMC adapt to meet cellular energy demands and support immune function following intramuscular LPS injection and clearance. The selected LPS dose may more closely align with a physiological stressor, such as manure cleanout within the facility while animals remain housed. Further investigation of increased LPS dosage and earlier sampling timepoint is warranted to further understand the effects of LPS on a cellular level and provide insight into stressors that may be seen in production as opposed to developing models that may not translate to industry situations.

## Data Availability

The raw data supporting the conclusions of this article will be made available by the authors, without undue reservation.
