# Supplementary material for: Metabolic and immune profiles of 1-year and 2.5+ year-old white leghorn roosters following intramuscular lipopolysaccharide injection
Source: Front Vet Sci. 2025 Feb 19;12:1547807. doi: 10.3389/fvets.2025.1547807 (PMC11880223; doi:10.3389/fvets.2025.1547807)
Supplement: Supplementary file 1 [file Table_1.docx]

Supplementary Material

# Supplementary Figures & Tables

## Supplementary Figures


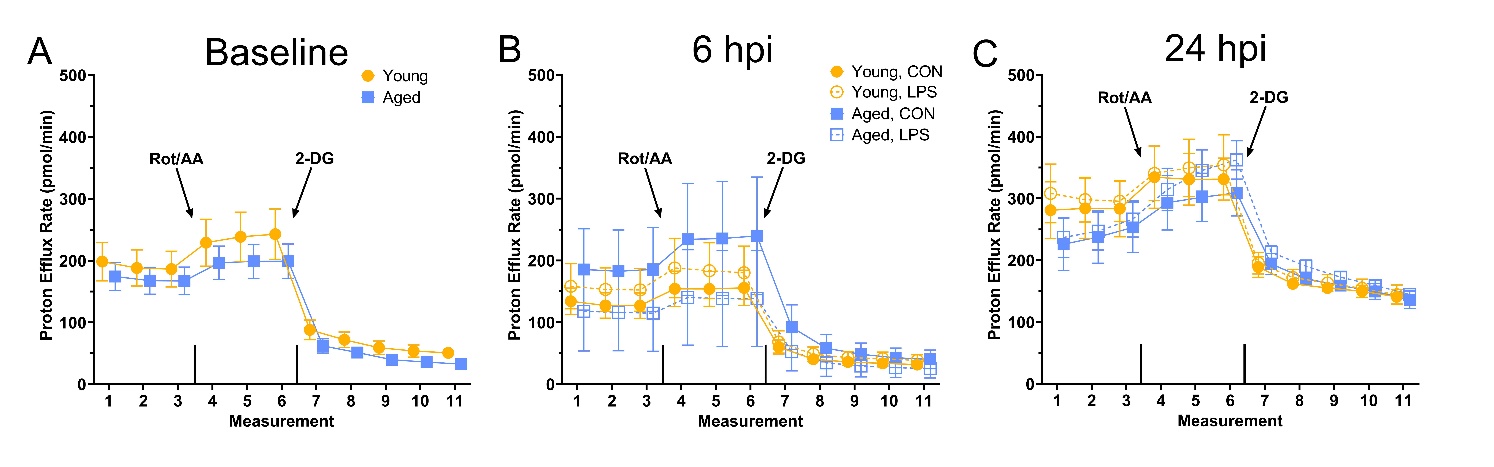


**Supplementary Figure 1.** The basal proton efflux rate (PER) potential of peripheral blood mononuclear cells from Young and Aged roosters ± 1 mg/kg intramuscular LPS injection at (**A**) baseline, (**B**) 6 hpi, and (**C**) 24 hpi. Data represent the mean ± SEM (n=12 young and 8 aged roosters at baseline, n=6 young and 4 aged roosters/treatment at 6 hpi**,** and n=6 young and 4 aged roosters/treatment at 24 hpi). No significant differences were found within any timepoint. This assay measures real-time glycolysis, including compensatory and residual glycolysis, by tracking extracellular acidification. After measuring basal PER and glycolysis, rotenone/antimycin A (Rot/AA) inhibits mitochondrial respiration, forcing a glycolytic shift to estimate compensatory glycolysis. This is followed by 2-deoxy-D-glucose (2-DG), which inhibits glycolysis, allowing differentiation of acidification sources beyond glycolysis and the TCA cycle.

**
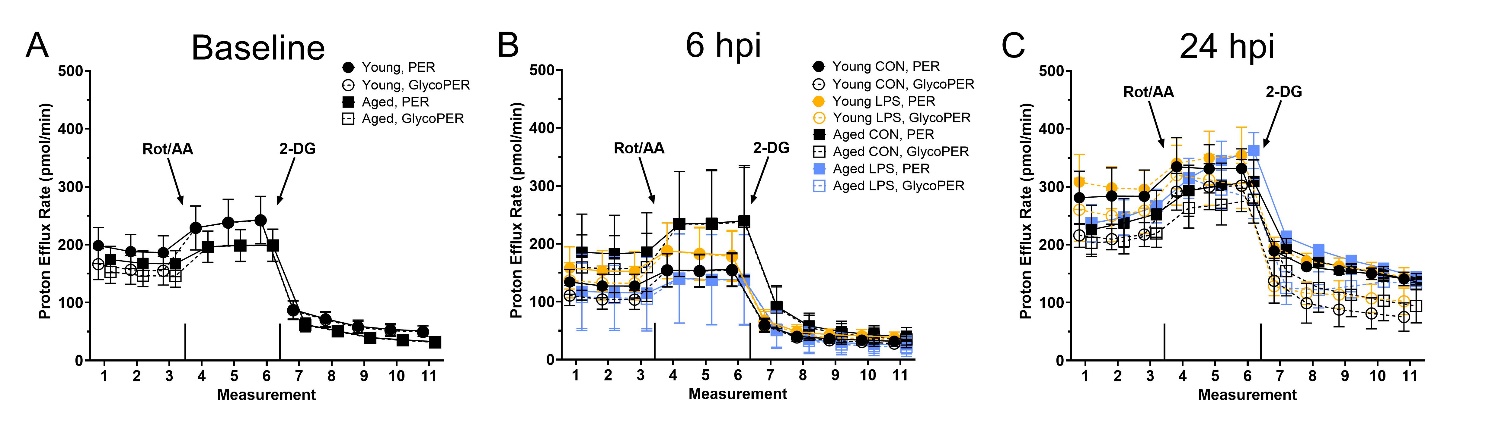
Supplementary Figure 2.** The basal proton efflux rate (PER) and glycolytic proton efflux rate (glycoPER) potential of peripheral blood mononuclear cells from Young and Aged roosters ± 1 mg/kg intramuscular LPS injection at (**A**) baseline, (**B**) 6 hpi, and (**C**) 24 hpi. Data represent the mean ± SEM (n=10 young and 8 aged roosters at baseline, n=6 young and 4 aged roosters/treatment at 6 hpi**,** and n=6 young and 4 aged roosters/treatment at 24 hpi). No significant differences were found within any timepoint. This assay measures real-time glycolysis, including compensatory and residual glycolysis, by tracking extracellular acidification. After measuring basal PER and glycolysis, rotenone/antimycin A (Rot/AA) inhibits mitochondrial respiration, forcing a glycolytic shift to estimate compensatory glycolysis. This is followed by 2-deoxy-D-glucose (2-DG), which inhibits glycolysis, allowing differentiation of acidification sources beyond glycolysis and the TCA cycle.

## Supplementary Tables

**Supplementary Table 1.** Basal proton efflux rate (PER), basal glycolysis, compensatory glycolysis, and post-2-DG acidification by rooster age ± 1 mg/kg intramuscular LPS injection at all timepoints. Data represents the mean ± SEM (n=12 young and 8 aged roosters at baseline, n=6 young and 4 aged roosters/treatment at 6 hpi**,** and n=6 young and 4 aged roosters/treatment at 24 hpi).

|  | Young | | Aged | | Adj. *P*-value | | |
| --- | --- | --- | --- | --- | --- | --- | --- |
| Measure (pmol/min) | CON | LPS | CON | LPS | Age | Trt^1^ | Age x Trt^1^ |
| Basal PER |  |  |  |  |  |  |  |
| Baseline | 186.2±25.6 | - | 167.0±26.9 | - | 0.61 | - | - |
| 6 hpi | 147.6±52.9 | 212.8±52.9 | 185.6±61.4 | 114.8±61.4 | 0.61 | 0.96 | 0.25 |
| 24 hpi | 283.3±35.8 | 295.5±35.8 | 253.1±41.5 | 266.7±41.5 | 0.46 | 0.74 | 0.98 |
| ΔBasal PER for 6 hpi | -31.9±61.4 | 11.2±61.4 | -3.4±53.0 | -34.7±53.0 | - | - | - |
| ΔBasal PER for 24 hpi | 82.7±41.2 | 84.0±41.2 | 64.1±44.7 | 117.2±44.7 | - | - | - |
| Basal glycolysis |  |  |  |  |  |  |  |
| Baseline | 154.8±21.8 | - | 145.9±23.0 | - | 0.78 | - | - |
| 6 hpi | 123.8±46.5 | 184.2±46.5 | 159.4±53.9 | 105.3±53.9 | 0.67 | 0.95 | 0.27 |
| 24 hpi | 217.4±28.6 | 258.6±28.6 | 219.2±33.2 | 266.7±33.2 | 0.87 | 0.17 | 0.92 |
| ΔBasal glycolysis for 6 hpi | -25.8±52.5 | 20.5±52.5 | -3.6±47.4 | -27.0±47.4 | - | - | - |
| ΔBasal glycolysis for 24 hpi | 63.2±35.0 | 77.8±35.0 | 56.2±32.9 | 134.4±32.9 | - | - | - |
| Compensatory glycolysis |  |  |  |  |  |  |  |
| Baseline | 242.3±34.6 | - | 199.5±36.4 | - | 0.41 | - | - |
| 6 hpi | 179.5±71.3 | 273.7±71.3 | 239.5±82.8 | 140.6±82.8 | 0.64 | 0.98 | 0.23 |
| 24 hpi | 312.3±39.3 | 331.07±39.3 | 283.7±45.6 | 314.5±45.6 | 0.60 | 0.57 | 0.89 |
| ΔCompensatory glycolysis for 6 hpi | -53.2±86.3 | 6.2±86.3 | 10.0±71.1 | -35.0±71.1 | - | - | - |
| ΔCompensatory glycolysis for 24 hpi | 57.9±48.9 | 52.1±48.9 | 54.2±48.9 | 138.9±48.9 | - | - | - |
| Post-2-DG acidification |  |  |  |  |  |  |  |
| Baseline | 48.5±7.7 | - | 28.9±8.2 | - | 0.10 | - | - |
| 6 hpi | 30.7±13.3 | 48.7±13.3 | 34.8±16.3 | 26.9±16.3 | 0.56 | 0.74 | 0.40 |
| 24 hpi | 71.5±23.1 | 90.0±23.1 | 92.1±26.8 | 100.0±26.8 | 0.55 | 0.61 | 0.83 |
| ΔPost-2-DG acidification for 6 hpi | -15.6±17.8 | -4.9±17.8 | 8.0±11.6 | -5.5±11.6 | - | - | - |
| ΔPost-2-DG acidification for 24 hpi | 11.9±15.6 | 58.1±15.6 | 65.4±24.9 | 69.3±24.9 | - | - | - |

Different letter superscripts within a timepoint are significantly different (Adjusted *P* ≤ 0.05).

^1^Trt = Injection main effect
